# Late Sodium Current in Human Atrial Cardiomyocytes from Patients in Sinus Rhythm and Atrial Fibrillation

**DOI:** 10.1371/journal.pone.0131432

**Published:** 2015-06-29

**Authors:** Claire Poulet, Erich Wettwer, Morten Grunnet, Thomas Jespersen, Larissa Fabritz, Klaus Matschke, Michael Knaut, Ursula Ravens

**Affiliations:** 1 Department of Pharmacology and Toxicology, Medical Faculty, TU Dresden, Dresden, Germany; 2 Danish Arrhythmia Research Centre, University of Copenhagen, Copenhagen, Denmark; 3 Centre for Cardiovascular Sciences, School of Clinical and Experimental Medicine, College of Medical and Dental Sciences, University of Birmingham, Birmingham, United Kingdom; 4 Clinic for Cardiac Surgery, Heart Center Dresden, Dresden, Germanymailto; Georgia State University, UNITED STATES

## Abstract

Slowly inactivating Na^+^ channels conducting “late” Na^+^ current (I_Na,late_) contribute to ventricular arrhythmogenesis under pathological conditions. I_Na,late_ was also reported to play a role in chronic atrial fibrillation (AF). The objective of this study was to investigate I_Na,late_ in human right atrial cardiomyocytes as a putative drug target for treatment of AF. To activate Na^+^ channels, cardiomyocytes from transgenic mice which exhibit I_Na,late_ (ΔKPQ), and right atrial cardiomyocytes from patients in sinus rhythm (SR) and AF were voltage clamped at room temperature by 250-ms long test pulses to -30 mV from a holding potential of -80 mV with a 100-ms pre-pulse to -110 mV (protocol I). I_Na,late_ at -30 mV was not discernible as deviation from the extrapolated straight line IV-curve between -110 mV and -80 mV in human atrial cells. Therefore, tetrodotoxin (TTX, 10 μM) was used to define persistent inward current after 250 ms at -30 mV as I_Na,late_. TTX-sensitive current was 0.27±0.06 pA/pF in ventricular cardiomyocytes from ΔKPQ mice, and amounted to 0.04±0.01 pA/pF and 0.09±0.02 pA/pF in SR and AF human atrial cardiomyocytes, respectively. With protocol II (holding potential -120 mV, pre-pulse to -80 mV) TTX-sensitive I_Na,late_ was always larger than with protocol I. Ranolazine (30 μM) reduced I_Na,late_ by 0.02±0.02 pA/pF in SR and 0.09±0.02 pA/pF in AF cells. At physiological temperature (37°C), however, I_Na,late_ became insignificant. Plateau phase and upstroke velocity of action potentials (APs) recorded with sharp microelectrodes in intact human trabeculae were more sensitive to ranolazine in AF than in SR preparations. Sodium channel subunits expression measured with qPCR was high for SCN5A with no difference between SR and AF. Expression of SCN8A and SCN10A was low in general, and lower in AF than in SR. In conclusion, We confirm for the first time a TTX-sensitive current (I_Na,late_) in right atrial cardiomyocytes from SR and AF patients at room temperature, but not at physiological temperature. While our study provides evidence for the presence of INa,late in human atria, the potential of such current as a target for the treatment of AF remains to be demonstrated.

## Introduction

The long-lasting plateau phase of the cardiac action potential is supported by L-type Ca^2+^ current [1], but slowly inactivating Na^+^ channels conducting “late” Na^+^ current (I_Na,late_) also contribute to the plateau phase and are related to ventricular arrhythmogenesis under pathological conditions [2–4]. Single channel studies have revealed that I_Na,late_ is conducted by cardiac Nav1.5 channels operating in special gating modes ([4]; for review see [5]) that clearly distinguish them from background Na^+^ currents.

In animal and human ventricular cells, I_Na,late_ is enhanced in heart failure [2–4], ischemia [6] or congenital disease (long QT syndrome 3, LQT3)[7]. In a mouse model of LQT3 (ΔKPQ), the increase in late Na^+^ current results in the prolongation of action potential (AP) duration in both ventricular and atrial tissues [8,9], suggesting that, in addition to its role in the ventricle, I_Na,late_ can also modulate atrial repolarization. In this context it is notable that LQT3 syndrome has been associated with familial atrial fibrillation (AF) [10–12], and a large proportion of SCN5A mutations found in young patients with lone AF actually increased I_Na,late_ [13]. A recent study reported the presence in human right atrial myocytes of late Na^+^ currents with larger amplitude in cells from patients with chronic AF [14]. This finding is of clinical interest, because enhancement of I_Na,late_ in AF could indeed contribute to the maintenance of the arrhythmia via increased Na^+^ influx during the AP plateau phase leading to Ca^2+^ overload via modulation of NCX activity[15,16]. Moreover, conventional antiarrhythmic drugs that block peak Na^+^ current (I_Na,peak_) are associated with considerable cardiac and extra-cardiac side effects including life-threatening arrhythmia. Therefore, I_Na,late_ might provide a useful novel target for treatment of AF if compounds that preferentially block I_Na,late_ over I_Na,peak_ can be developed. The anti-anginal drug ranolazine seems to provide this property, since it blocks I_Na,late_ with ~40-fold higher potency than I_Na,peak_ (i.e. IC_50_ values 5.9 μM for late [17] versus 244 μM for peak I_Na_ in ventricular cardiomyocytes [18]). Interestingly, experimental and clinical studies have shown that ranolazine has antiarrhythmic effects in both ventricles and atria (reviewed in [19]).

In previous preliminary experiments from our laboratory [20], using a repolarizing ramp pulse we detected I_Na,late_ only in the presence but not in the absence of the sea anemone toxin ATXII, known to delay inactivation of I_Na_ [21]. In addition, we also did not measure any I_Na,late_ at -30 mV as current amplitude deviating from the extrapolated straight line of background (“leak”) current-voltage relationship between -110 mV and -80 mV [20]. Therefore we validated our step voltage clamp protocols for the detection of I_Na,late_ in cardiomyocytes from ΔKPQ mice (LQT3). These mice have a knock-in gain of function mutation in SCN5A, the gene coding for the major cardiac sodium channel Nav1.5, which leads to increased I_Na,late_ in both ventricular and atrial myocytes [8,22]. We then searched for the presence of I_Na,late_ as TTX-sensitive current in atrial myocytes from patients in sinus rhythm (SR) and with chronic AF, and studied the impact of the putative preferential I_Na,late_ blocker ranolazine on the shape of right atrial APs. Some of the results have been published in preliminary form [23].

## Materials and Methods

### Human tissue samples and patient’s characteristics

The study conforms with the Declaration of Helsinki and was approved by the ethics committee of Dresden University of Technology (No. EK790799). Each patient gave written, informed consent. Right atrial appendages were obtained from patients with SR and with chronic AF (AF > 6 months). More information on patients is given in Table 1.

**Table 1:** Clinical characteristics of patients

	SR	AF
	n = 23	n = 17
*General demographics*		
Gender [m/f]	19/4	12/5
Age [years]	64.4 ± 1.8	71.9 ± 1,6**
BMI [kg/m2]	29.8 ± 1.0	26.9 ± 1.0
Hypertension, n	20 (87.0)	17 (100.0)
Diabetes mellitus, n	8 (34.8)	8 (47.1)
Hyperlipidaemia, n	15 (65.2)	12 (70.1)
CAD, n	19 (82.6)	0
AVD/MVD, n	4 (17.4)	12 (70.1)
CAD + AVD/MVD, n	0	5 (29.4)
*Hemodynamic parameters*		
LVEF [%]	50.6 ± 3.0	48.2 ± 4.0
LA [mm]	41.5 ± 1.7	49.6 ± 2.3**
LVEDD [mm]	51.2 ± 2.5	55.7 ± 1,9
LVWTED [mm]	12.2 ± 0.6	11.9 ± 0.4
*Cardiovascular medication* (n)		
Digitalis (%)	0	7 (41.2)
ACE inhibitors (%)	14 (60.9)	11 (64.7)
AT1 blockers (%)	6 (26.1)	0
β-blockers (%)	20 (87.0)	15 (88.2)
Calcium channel blockers (%)	5 (21.7)	4 (23.5)
Diuretics (%)	7 (30.4)	13 (76.5)
Nitrates (%)	0	0
Lipid-lowering drugs (%)	16 (70.0)	9 (52.9)

Abbreviations: ACE, angiotensin converting enzyme; AT, angiotensin receptor; AVD, aortic valve disease; BMI, body mass index; CAD, coronary artery disease; LA, left atrial diameter; LVEDD, left ventricular end-diastolic diameter; LVEF, left ventricular ejection fraction; LVWTED, left ventricular wall thickness at end of diastole; MVD, mitral valve disease; n, number of patients

* p<0.05 Student’s unpaired t-test for continuous variables and [χ]^2^ test for categorical variables.

### ΔKPQ-SCN5A mice

Animal care and experimental protocols were conducted in accordance with the German federal animal protection law and approved by the institutional committee at the Technical University Dresden. The investigation conforms to the Guide for the Care and Use of Laboratory Animals published by the US National Institutes of Health (NIH Publication No. 85–23, revised 1996). Experiments and analysis performed in adult littermate wild-type (WT) and heterozygous ΔKPQ mice [9], 17–19 weeks old, were blinded to genotype (group A and B). Upon unblinding at the end of the analysis, group A turned out to be ΔKPQ, and group B was WT.

### Electrophysiological recordings

Mice were sacrificed by cervical dislocation under CO_2_ anesthesia. Mouse and human cardiomyocytes were isolated as previously described [24,25]. The conventional voltage-clamp technique in the ruptured patch configuration was used to measure ion currents. Experiments were performed at room temperature, unless otherwise stated, in the following bath solution (mM): NaCl 120, HEPES 10, MgCl_2_ 1, CsCl 10, glucose 10 and CaCl_2_ 0.5 (pH 7.4, adjusted with CsOH). The pipette solution (pH 7.2, adjusted with CsOH) consisted of (mM): NaCl 5, caesium methanesulfonate 90, CsCl 20, HEPES 10, Mg-ATP 4, Tris-GTP 0.4, EGTA 10 and CaCl2 3, with calculated free Ca2+ concentration of 60 nM (computer program EQCAL, Biosoft, Cambridge, UK). Experiments were done in the presence of 1μM nisoldipine to block L-type Ca^2+^ currents. After forming a giga-ohm seal and breaking into the cell, capacitance was measured with small hyperpolarizing clamp steps from -40 mV to -42 mV. Mean values were 70.4±3.3 pF (53/23, cells/patients) in SR and 84.5±4.4 pF (54/17) in AF myocytes (P<0.012, unpaired, two-tailed t-test). Mean cell capacitances for murine ventricular cardiomyocytes were 101 ± 7.1 pF (15/3) in WT and 96.7 ± 4.6 pF (33/5, difference n.s.) in ΔKPQ mice. The respective capacitances for atrial cardiomyocytes were 35.3 ± 2.8 pF (9/3) and 40.4 ± 4.1 pF (8/3; n.s.).

### Voltage protocols

**Fig 1:**
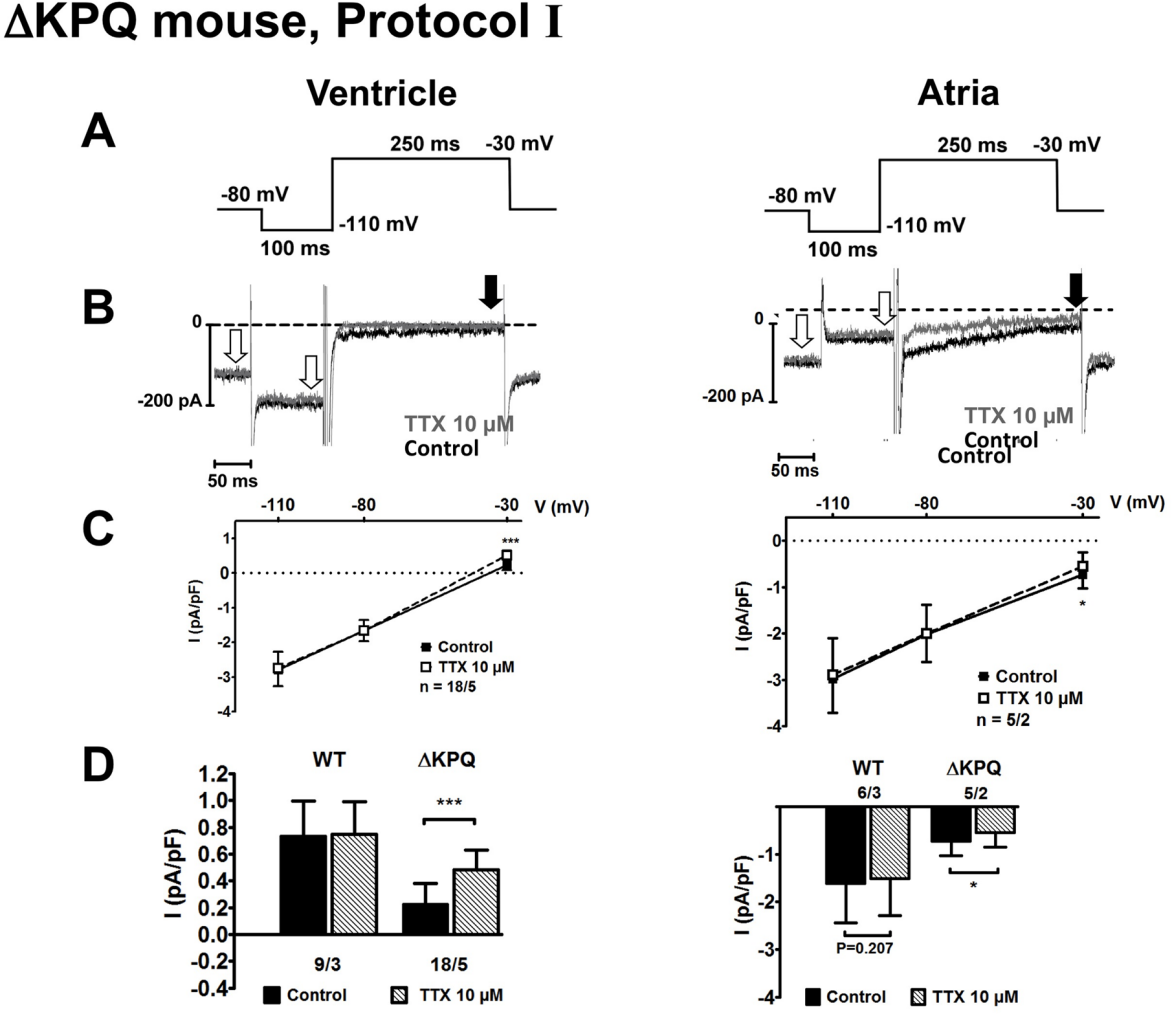
Effects of tetrodotoxin on I_Na,late_ in ventricular and atrial cardiomyocytes (CM) from ΔKPQ-SCN5A mice. **A** and **B**: Voltage clamp protocol Ⅰ and examples of original current tracings recorded in ΔKPQ mouse myocytes under control conditions or in the presence of 10 μM tetrodotoxin (TTX). Late sodium currents were measured at the end of the 250 ms-long pulse at -30 mV (black arrow). As internal control, current amplitude was also measured at -80 mV and -110 mV (white arrows). **C**: Current densities were plotted for each voltage without (control, black circles) and after addition of 10 μM TTX (open squares). **D**: Currents measured at -30 mV before (black columns) and after TTX exposure (grey columns) in myocytes from wild type (WT) and ΔKPQ mice. Numbers below the columns (x/y) indicate number of cells per number of animals. TTX-sensitive current expressed as current density (in pA/pF). **P < 0.01; ***P < 0.001; paired Student’s t-test.

In protocol I [20] the cells were held close to the physiological resting membrane potential at -80 mV, a 100 ms-long step to -110 mV was used to increase availability of Na^+^ channels, and Na^+^ channels were activated at –30 mV (Fig 1A), giving rise to a huge peak inward current (off-scale in Fig 1B), the major fraction of which rapidly inactivated within ~10 ms and then declined very slowly until the end of the 250-ms pulse at -30 mV. Full activation of I_Na,peak_ at normal Na^+^ concentration is likely to cause escape of voltage control due to the limitations of the patch-clamp amplifier. In order to reduce this problem we used in protocol II a short (5 ms) activating step to +50 mV (close to the reversal potential of I_Na,peak_ to avoid loss of voltage control) before stepping to –30 mV to observe late Na^+^ current, as suggested by Sossalla et al.[14]. The cells were held at -120 mV for near complete Na^+^ channel availability, and for comparison with protocol I a 100-ms step to -80 mV between V_h_ and activating step was interpolated in the majority of cells. When possible, both protocols were applied to the same cell successively, and late Na^+^ currents were measured 50 ms and 250 ms after Na^+^ channels activation at -30 mV. Current amplitude was also measured at -110 and -80 mV, and at -120 and -80 mV, respectively, for quality control over time: if the difference in current amplitude after drug application was larger than 10 pA at -80 mV (or >20 pA at -120 mV for cells without a step to -80 mV), the experiment was excluded from statistical analysis.

### Action potential recordings

Action potentials (APs) were recorded with standard intracellular microelectrodes in human right atrial trabeculae paced at a frequency of 1 Hz [26]. The bath solution contained (in mM): NaCl 127, KCl 4.5, MgCl_2_ 1.5, CaCl_2_ 1.8, glucose 10, NaHCO_3_ 22, NaH_2_PO_4_ 0.42, equilibrated with O_2_-CO_2_ [95%:5%] at 36.5 ± 0.5°C, pH 7.4. Preparations were regularly stimulated at 1 Hz for at least 1 hour before data acquisition with a custom-made computer program (University of Szeged, Hungary) that also generated electrical stimuli. The following AP parameters were analysed using the LabChart software (ADInstruments, Spechbach, Germany): resting membrane potential (RMP), action potential amplitude (APA) and duration at 20%, 50% and 90% of repolarization (APD_20_, APD_50_ and APD_90_), plateau potential defined as the mean potential (mV) in the time window between 20% of APD_90_ plus 5 ms (PLT_20_), and maximum upstroke velocity (dV/dt_max_).

### Expression of sodium channel subunits

Quantification of mRNA transcripts encoding for sodium channel subunits was performed on right atrial appendage from 10 SR (mean age 75.5±1.3 years, 4 females) and 14 AF (mean age 77.2±1.1 years, 7 females) patients. Total RNA extraction, cDNA synthesis and quantitative real-time PCR (qPCR) was performed as previously described [27]. The following pre-designed gene expression assays from Applied Biosystems were used for quantification; SCN1A (Hs00374696_m1), SCN2A (Hs00221379_m1), SCN3A (Hs00366902_m1), SCN4A (Hs01109480_m1), SCN5A (Hs00165693_m1), SCN8A (Hs00274075_m1), SCN10A (Hs01045137_m1), SCN1B (Hs00962350_m1). The primers and probes targeting SCN2B, SCN3B, and SCN4B were custom designed and synthesized by Applied Biosystems, following submission of intron spanning sequences using Primer Express 3.0 software. PPIA (Assay Hs99999904_m1) was used for normalization.

### Chemicals and Drugs

Tetrodotoxin was purchased from Carl Roth (Karlsruhe, Germany). All other compounds were from Sigma-Aldrich (Steinheim, Germany).

### Statistical analysis

Data are expressed as means ± SEM. Differences between data were compared by paired or unpaired Student’s *t* test (with Welch’s correction if indicated). P<0.05 was considered statistically significant.

## Results

### Effects of tetrodotoxin on I_Na,late_ in cardiomyocytes from ΔKPQ mice

In order to test whether the clamp protocol for testing persisting membrane current at -30 mV as deviation from the extrapolated straight line between -110 mV (-120 mV) and -80 mV is sensitive enough for detecting I_Na,late_, we studied cardiomyocytes from ΔKPQ mice in which I_Na,late_ has been detected previously [8,22]. In addition we also used a slightly modified protocol with which I_Na,late_ has been studied in human atrial cardiomyocytes [14] and applied tetrodotoxin (TTX, 10 μM) to test for contribution of Na^+^ channels. Fig 1 shows currents measured in ventricular and atrial cells from ΔKPQ mice (Fig 1B–1D) and also from wild type (WT) mice (Fig 1D) with protocol I. At -110 mV and at -80 mV, current amplitudes were stable in the presence of TTX. At -30 mV control currents were outwardly directed in ventricular myocytes, possibly due to residual I_K1_, whereas in atrial cells they were inwardly directed due to smaller I_K1_/I_leak_ ratio. At -30 mV, TTX clearly unmasked a small, slowly decaying inward current. Current amplitude 250 ms after Na^+^ channel activation was indeed significantly more outward in ventricular cells and significantly less inward in atrial cells upon application of 10 μM TTX, consistent with block of an inward current, which is conducted via Na^+^ channels and probably represents I_Na,late_. Please note, that after addition of TTX, current amplitudes at -30 mV no longer deviated from a straight line, clearly indicating the presence of a linear conductance or background current (Fig 1C). In contrast, no effect of TTX was observed in cells from WT mice (Fig 1D). The amplitudes of TTX-sensitive current density determined as difference current measured with each protocol (Fig 1D) were 0.27 ± 0.06 pA/pF in ventricular cells (n = 18 cells from 5 ΔKPQ animals; 18/5) with protocol I, and 0.63 ± 0.10 pA/pF (n = 15/4; p<0.05) when protocol II was used (see S1 Fig), the difference being most likely due to the more negative holding potential and the subsequently larger fraction of Na^+^ channels available for activation with protocol II.

Although TTX significantly reduced late Na^+^ currents (I_Na,late_) also in atrial cardiomyocytes from ΔKPQ but not WT mice, the difference in TTX-sensitive current between WT and ΔKPQ atrial cells failed to reach the level of statistical significance with either protocol (S6 Fig), probably due to the small sample size. Nevertheless, with protocol II TTX-sensitive current amplitudes in ΔKPQ atrial cells were significantly larger than with protocol I, i.e. 0.50 ± 0.12 pA/pF (n = 4/3) with protocol II, and 0.18 ± 0.05 pA/pF (n = 5/2; p = 0.030) with protocol I.

### Effects of tetrodotoxin on I_Na,late_ in human atrial myocytes

**Fig 2:**
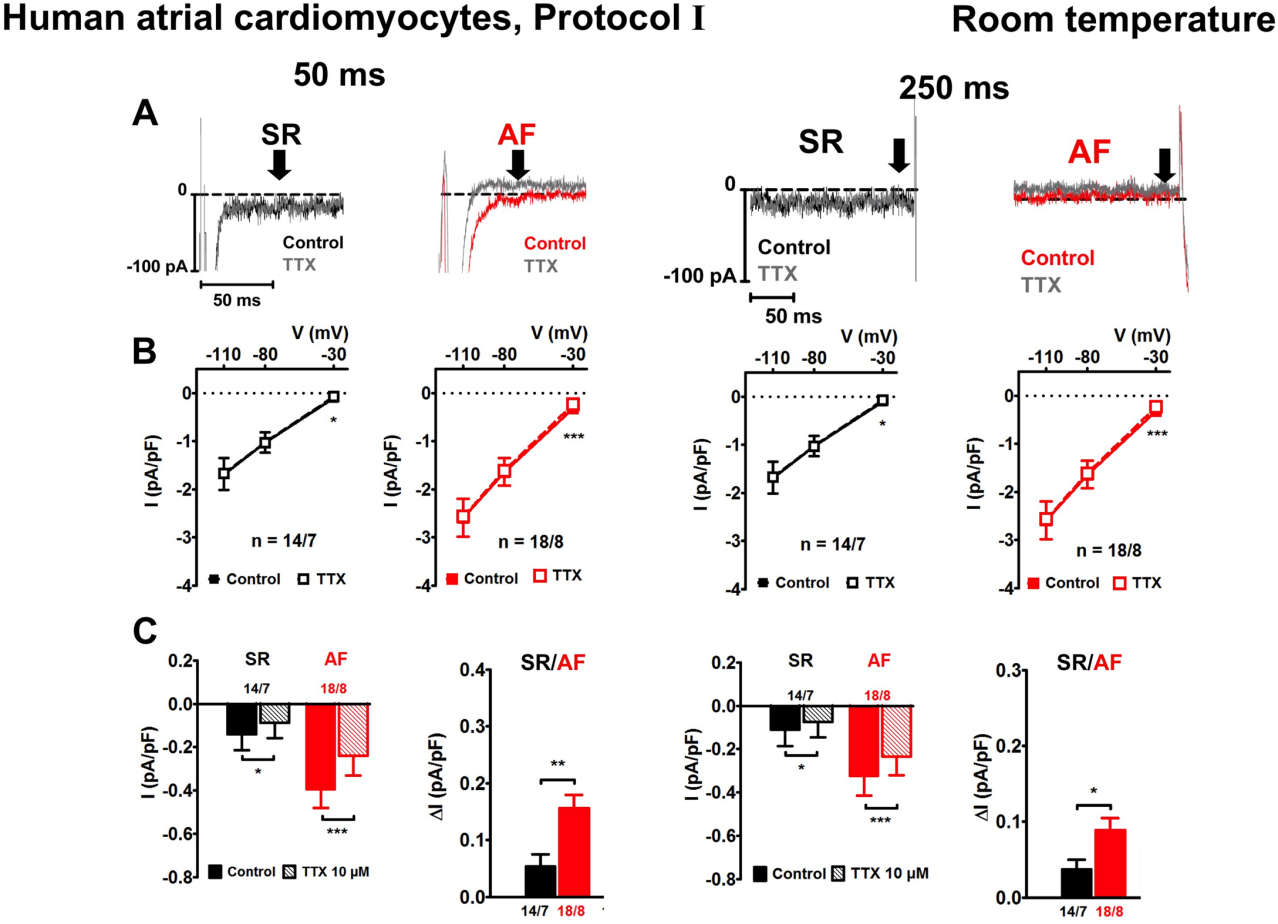
Effects of tetrodotoxin on I_Na,late_ in human atrial myocytes from patients in sinus rhythm (SR, black) and atrial fibrillation (AF, red) 50 ms and 250 ms after Na^+^ channel activation. **A** Examples of original current tracings (top) measured at room temperature with protocol Ⅰ, under control conditions and in the presence of 10 μM TTX. Late sodium currents were measured 50 ms (left side) 250 ms (right side) after the beginning of the pulse at -30 mV (black arrow). **B**: Current density plotted for each voltage yielded a straight line after addition of TTX. **C**: Effect of 10 μM TTX on currents expressed as current density (in pA/pF) at -30 mV in SR and AF cardiomyocytes, and *P < 0.05, ***P < 0.001; paired Student’s t-test (**B, C**) for currents before and after exposure to TTX, or unpaired Student‘s t test with Welch’s correction for TTX-sensitive current in SR and AF.

As both protocols allowed the detection of late Na^+^ currents in ΔKPQ cardiomyocytes, we performed similar experiments in human atrial myocytes from patients in sinus rhythm (SR) or chronic atrial fibrillation (AF). Currents were analysed at room temperature 50 ms and 250 ms (Fig 2) after Na^+^ channel activation. Examples of complete current tracings are given in S2 Fig As for the murine cardiomyocyte experiments, stability of current amplitude at -80 mV (or -120 mV, see section 2.5) was used as quality control, and recordings with a shift >10 pA at -80 mV (or >20 pA at -120 mV) after addition of TTX were discarded. In cells from one patient in SR, we measured a surprisingly robust late Na^+^ current with both protocols. This particular patient had a prolonged QTc interval preoperatively (unpublished data) and was therefore excluded from further analysis.

At room temperature, TTX clearly decreased mean current amplitude after 250 ms at -30 mV in SR and AF cardiomyocytes (Fig 2A–2C). The amplitude of TTX-sensitive I_Na,late_ measured in AF cells was significantly larger than in SR cells (Fig 2D): 0.09 ± 0.02 pA/pF in AF (n = 18/8) vs 0.04 ± 0.01 pA/pF in SR (n = 14/7, P = 0.021) with protocol I; and 0.18 ± 0.04 pA/pF in AF (n = 11/7) vs 0.07 ± 0.03 pA/pF in SR (n = 12/6, P = 0.013) with protocol II (see S3 Fig). These differences were even more prominent when currents were analysed earlier in the clamp step, i.e. after 50 ms (Fig 2, S3 Fig).

**Fig 3:**
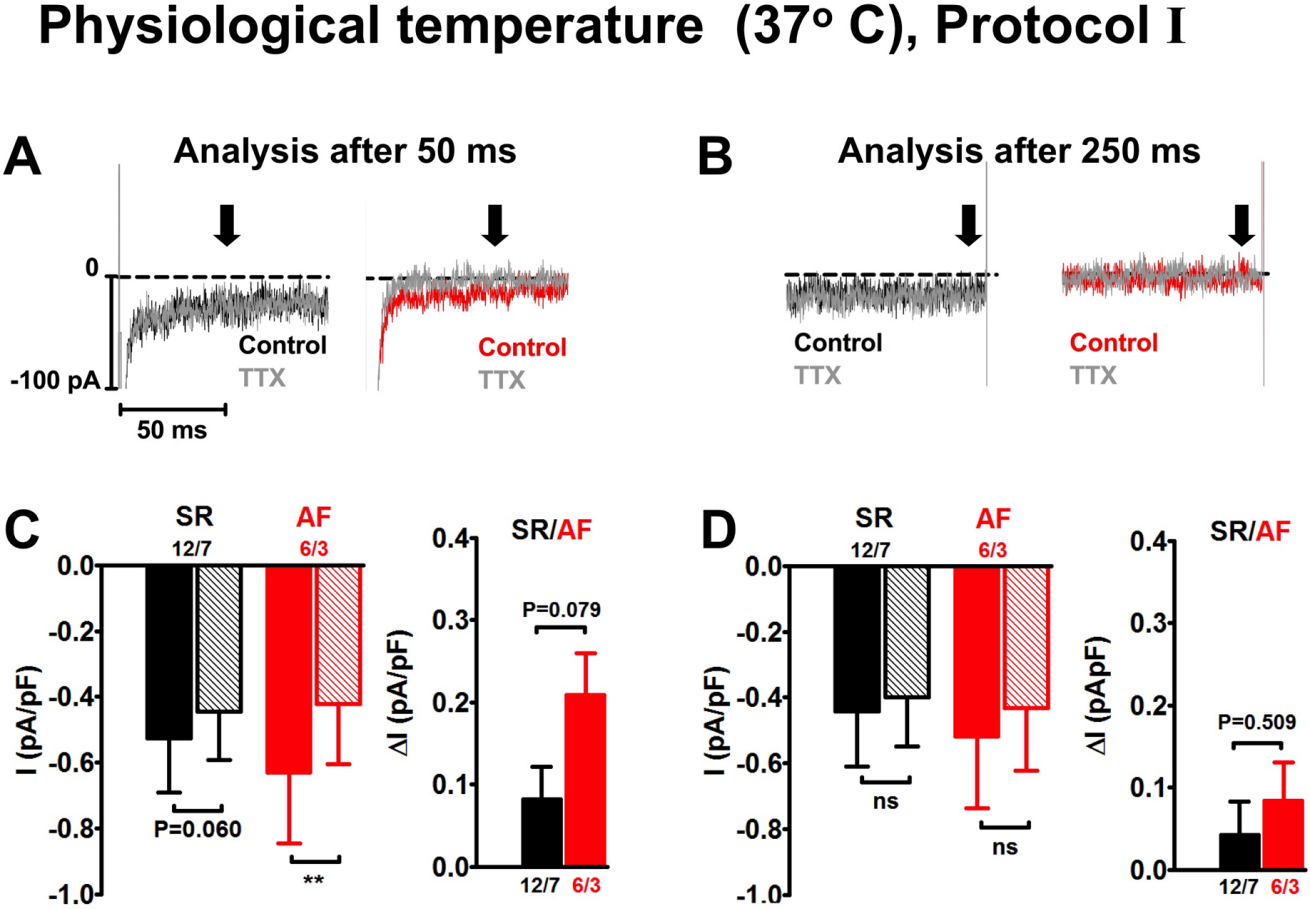
Effects of tetrodotoxin on I_Na,late_ measured at physiological temperature (37°C) with protocol I in human atrial myocytes 50 ms (A, C) and 250 ms (B, D) after Na^+^ channel activation. Similar lay-out as in Fig 2, but without I/V curves.

We performed additional experiments at 37°C to test whether I_Na,late_ amplitude was increased at physiological temperature (Fig 3). Only protocol I could be used because with a holding potential of -120 mV, recordings were too unstable for analysis. TTX significantly reduced current at -30 mV after 50 ms in AF but not in SR cells (S3A and S3C Fig). TTX-sensitive currents after 50 ms were 0.08 ± 0.04 pA/pF in SR (n = 12/7) and 0.21 ± 0.05 pA/pF in AF (n = 6/3), the difference between SR and AF just failing to reach the level of statistical significance (P = 0.079). After 250 ms no significant TTX effects were detected (S3B and S3D Fig) neither in SR nor AF, most likely due to a faster inactivation of the Na^+^ channels at physiological temperature.

### Effects of ranolazine on I_Na,late_ in human atrial myocytes

**Fig 4:**
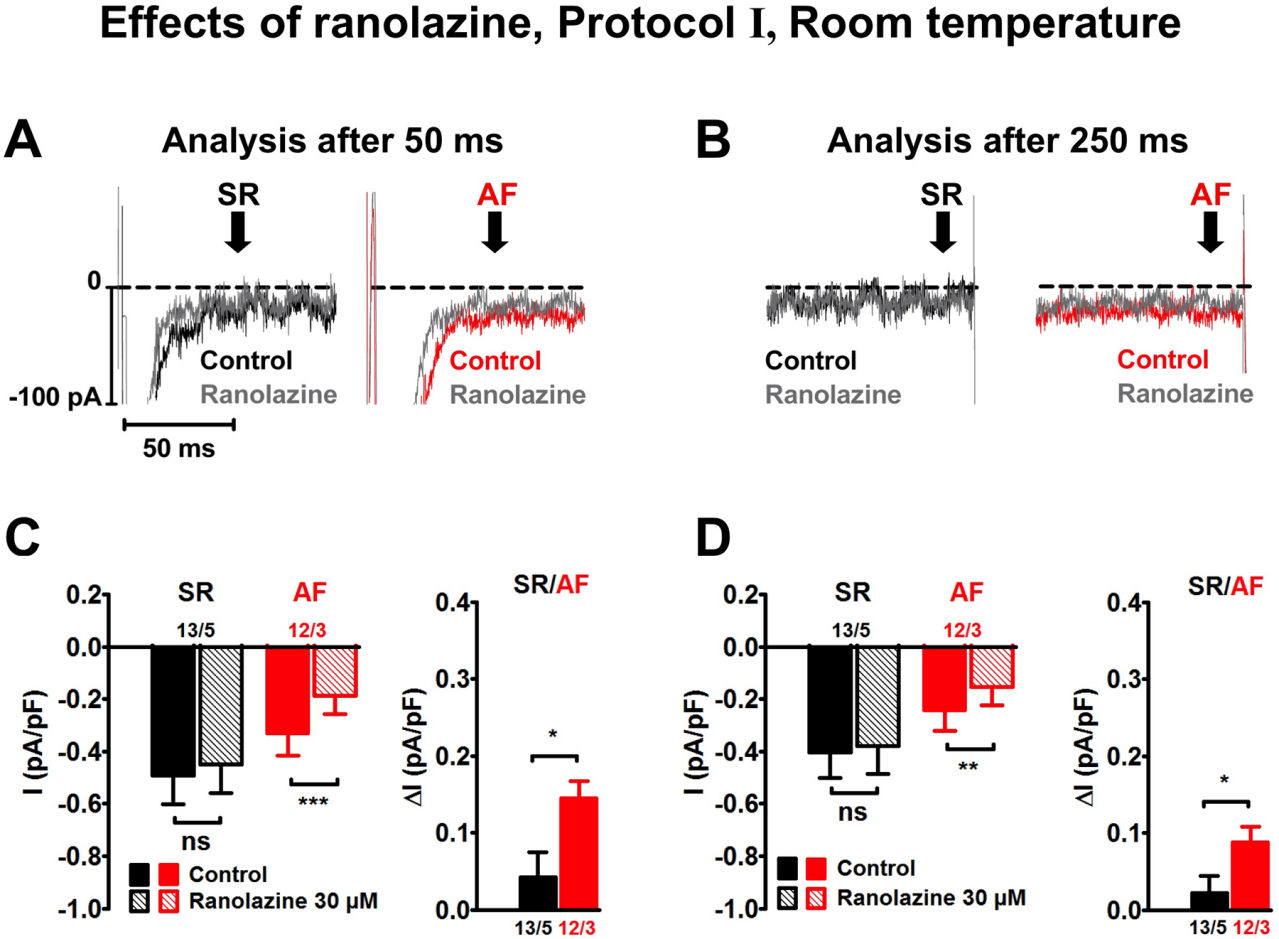
Effects of ranolazine on I_Na,late_ measured with protocol I in human atrial myocytes at room temperature. Similar lay-out as in Fig 3.

The effect of ranolazine (30 μM) on I_Na,late_ was studied at room temperature (Fig 4). Only protocol I was used in those experiments, and we found that ranolazine significantly reduced current amplitude only in AF myocytes (Fig 4B and 4C). The amplitude of ranolazine-sensitive late Na^+^ current density after 50 ms was larger in AF than in SR cells, i.e. 0.15 ± 0.02 pA/pF (n = 12/3) and 0.04 ± 0.03 pA/pF (n = 13/5; P<0.05), respectively. After 250 ms, I_Na,late_ amplitude measured as ranolazine-sensitive current density was smaller in both SR and AF (Fig 4C), but still significantly larger in AF (0.09 ± 0.02 pA/pF) than SR cells (0.02 ± 0.02 pA/pF; P<0.05). The AF values were similar to TTX-sensitive currents described in section 3.1.

### Effects of ranolazine on human right atrial action potentials

**Fig 5:**
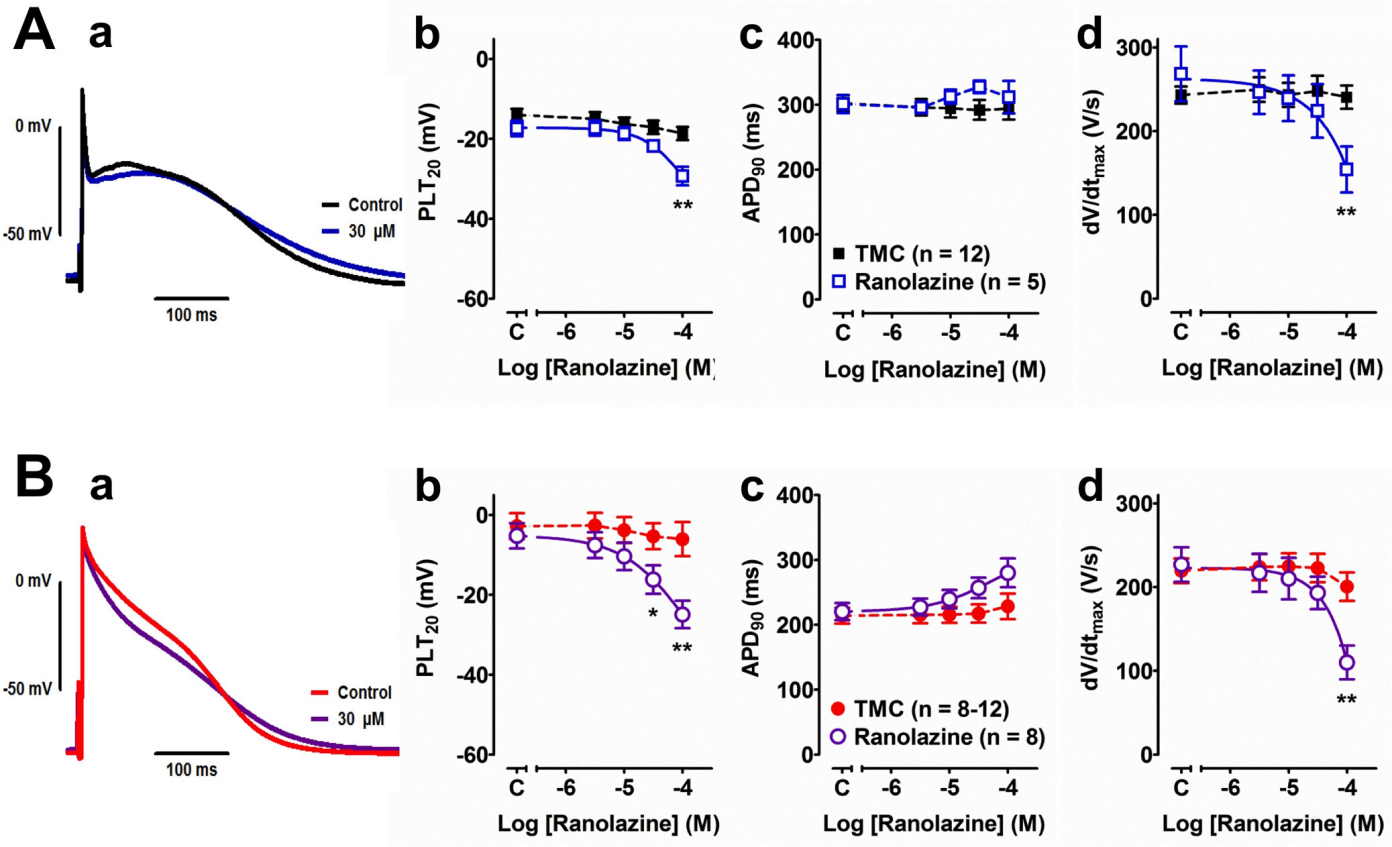
Effects of ranolazine on human right atrial action potentials. Action potentials were recorded in tissue from patients in SR (**Aa**) and in AF (**Ba**) under control conditions and after exposure to increasing concentrations of ranolazine. Scale bar: from 0 to -80 mV in the y axis; 100 ms in the x axis. **Ab-d** and **Bb-d**: Ranolazine effects on (blue and purple), compared to spontaneous changes in time-matched control experiments (TMC) without any drug added in plateau potential (mean potential at 20% of APD_**90**_ plus 5 ms, PLT_**20**_, **Ab, Bb**), on action potential duration at 90% of repolarization (APD_**90**_, **Ac, Bc**), and maximum upstroke velocity (dV/dt_**max**_, **Ad, Bd**). *P < 0.05; **P < 0.01; unpaired Student‘s t test.

In order to estimate the functional importance of I_Na,late_ for atrial action potentials (APs) right atrial trabeculae from patients in SR and AF were studied in the presence of cumulatively increasing concentrations of ranolazine (3–100 μM, 20 min at each concentration). The drug effects on all AP parameters are summarised in Tables 2 and 3. In the high concentration range, ranolazine reduced the plateau potential (PLT_20_) and depressed upstroke velocity dV/dt_max_ when compared to the pre-drug control values, and these effects were more prominent, and occurred at lower concentrations, in AF than SR trabeculae (see Tables 2 and 3). In AF but not in SR trabeculae, ranolazine prolonged APD_90_ at all concentrations and reduced APA at or >10 μM. The stability of AP parameters over time was studied in the absence of any drug (time-matched controls, TMC). PLT_20_ and dV/dt_max_ tended to decrease, and APD_90_ remained constant over time (Fig 5). Compared with TMC, 30 μM ranolazine significantly reduced plateau potential (PLT_20_) in AF trabeculae, and the effect of 100 μM ranolazine was significant in both SR and AF preparations (Fig 5). The prolongation of APD_90_ in AF trabeculae (Table 3) was not significant when compared to corresponding TMC values (Fig 5). With 100 μM ranolazine, dV/dt_max_ was considerably reduced, so that analysis and comparison of other parameters was impossible because some preparations failed to respond to electrical stimulation. Multicellular, superfused preparations usually require higher drug concentrations than single cardiomyocytes because of non-homogeneous drug distribution within the tissue.

**Table 2:** Effects of ranolazine on human right atrial action potential parameters (1 Hz) from patients in sinus rhythm (SR), n = 6.

AP parameter		Ranolazine			
	Pre-drug control	3 μM	10 μM	30 μM	100 μM
APD_90_ (ms)	265.8 ± 23.0	253.4 ± 26.1	261.7 ± 27.1	237.2 ± 31.4	255.5 ± 34.0
APD_50_ (ms)	128.8 ± 18.8	112.0 ± 22.7	110.5 ± 22.2	55.4 ± 20.7*	88.7 ± 26.4
APD_20_ (ms)	5.4 ± 0.8	4.6 ± 0.5	4.8 ± 0.6	5.3 ± 1.0	14.4 ± 6.5
PLT_20_ mV	-19.3 ± 1.3	-20.2 ± 1.7	-20.8 ± 2.1	-27.6 ± 2.1*	-33.8 ± 5.1*
APA (mV)	91.5 ± 1.8	94.6 ± 3.1	92.3 ± 3.0	88.6 ± 2.9	66.0 ± 11.2
RMP (mV)	-73.5 ± 1.0	-73.6 ± 0.8	-73.3 ± 1.0	-72.8 ± 0.8	-70.2 ± 1.1
dV/dt_max_ (V/s)	238.7 ± 12.8	274.6 ± 30.0	251.8 ± 32.6	221.0 ± 31.4	126.2 ± 26.9**

*P<0.05,

** P<0.01;

***P<0.001, versus pre-drug control

**Table 3:** Effects of ranolazine on human right atrial action potential parameters (1 Hz) from patients in atrial fibrillation (AF), n = 7.

AP parameter		Ranolazine			
	Pre-drug control	3 μM	10 μM	30 μM	100 μM
APD_90_ (ms)	214.3 ± 13.5	221.3 ± 13.4***	233.7 ± 14.9***	250.3 ± 16.7**	267.9 ± 22.0*
APD_50_ (ms)	97.9 ± 7.7	97.3 ± 7.9	97.9 ± 8.9.	94.0 ± 8.5	102.7 ± 14.5
APD_20_ (ms)	23.1 ± 2.9	22.1 ± 2.6	21.9 ± 2.4	20.9 ± 2.6	25.0 ± 3.0
PLT_20_ mV	-7.4 ± 2.6	-9.3 ± 3.1*	-12.2 ± 3.3***	-17.2 ± 3.1***	-23.6 ± 3.9***
APA (mV)	103.3 ± 1.1	103,3 ± 1.2	100.5 ± 1.5*	94.9 ± 2.1*	80.7 ± 7.0*
RMP (mV)	-79.0 ± 0.7	-78.4 ± 0.5	-78.1 ± 0.6	-77.9 ± 1.1	-76.1 ± 1.8
dV/dt_max_ (V/s)	233.4 ± 19.3	225.7 ± 24.1	197.1 ± 24.5**	188.1 ± 17.5*	114.7 ± 25.0***

*P<0.05,

** P<0.01;

***P<0.001, versus pre-drug control

### Expression of sodium channel subunits

**Fig 6:**
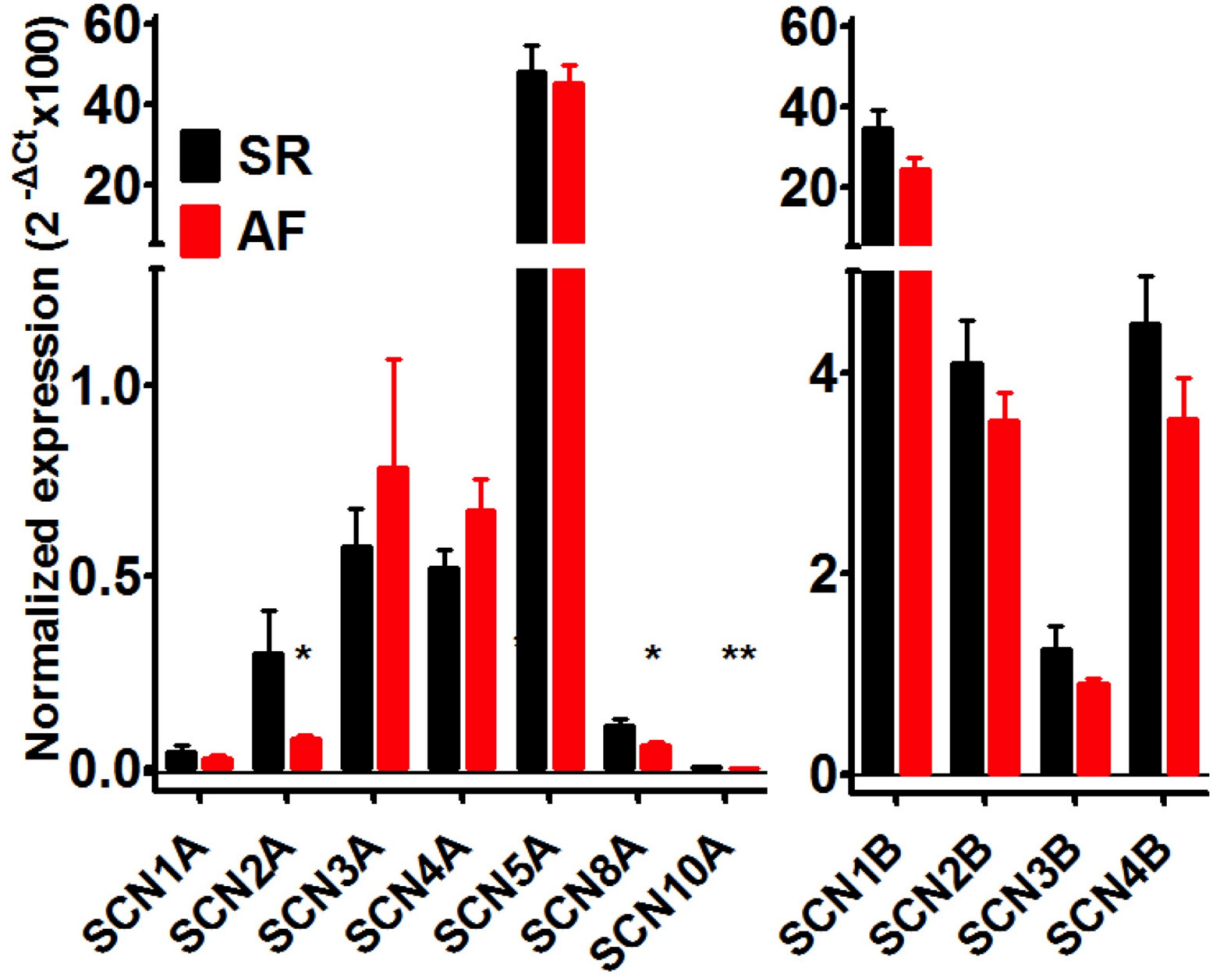
Expression of sodium channel subunits in right atrial tissue from patients in SR and AF. mRNA expression of various Na^**+**^ channel alpha- (left) and beta- (right) subunits was quantified by qPCR in 10 and 14 samples of patients in SR and AF respectively. *p < 0.05; unpaired Student‘s t test.

Recently, cardiomyocytes were shown to express also other Na^+^ channel subunits in addition to the cardiac specific Nav1.5 [28]. Therefore, the expression of various α- and β- subunits was quantified by qPCR in 10 SR and 14 AF samples (Fig 6). As expected, the major pore-forming α-subunit expressed in both SR and AF tissues was Nav1.5 (SCN5A). The TTX-sensitive channels SCN1A-4A were expressed at around 100-fold lower levels, which is in line with previous published data on non-diseased human atria [28]. The neuronal Na^+^ channel isoform Nav1.6, encoded by SCN8A, is upregulated in a rat model of heart failure and was suggested to contribute to I_Na,late_ [29]. This transcript was also expressed in human atrial tissue. The SCN10A transcript, encoding for Nav1.8, which was recently shown to be associated with slowing of atrioventricular conduction [30,31], exhibited an expression level approximately 10.000-fold lower than SCN5A. Though expressed at low levels only SCN2A, SCN8A and SCN10A showed significantly lower expression in AF than in SR. The SCN1B transcript encoding the Na^+^ channel β-subunit Navβ1 was expressed at high levels, SCN2B and SCN4B at intermediate levels and SCN3B at fairly low levels, but expression of these transcripts did not differ between SR and AF tissue.

## Discussion

The main findings of our present study were: (i) confirmation of a very small I_Na,late_ as TTX-sensitive current at room temperature, but not at physiological temperature, in human right atrial cardiomyocytes with larger TTX-sensitive current in cells from AF than SR patients; (ii) more prominent ranolazine effects on plateau potential and upstroke velocity in AF than SR preparations, and (iii) lack of correlation between I_Na,late_ amplitude and expression of Nav1.x α- and β-channel subunits in SR and AF tissue.

### I_Na,late_ in ventricular and atrial cardiomyocytes from ΔKPQ mice

One difficulty in detecting I_Na,late_ with standard rectangular clamp pulses is to discriminate between “leak” currents and current flow through non-inactivating channels due to technical limitations of the patch clamp technique. The seal resistance may not be sufficiently high to minimize “leak” current unrelated to currents flowing through ion channels. These leak currents have a linear current-voltage relationship that crosses the abscissa at the junction potential. Assuming a typical seal resistance of ~1 GΩ, leak current at a test potential of -30 mV is expected to be ~30 pA according to Ohm’s law. I_Na,late_ should become evident at the potential of maximum Na^+^ channel activation, i.e. at ~-30 mV. However, in preliminary work with K^+^ currents minimized by substitution of Cs^+^ for K^+^ and L-type Ca^2+^ current blocked by exposing the cells to 1 μM nisoldipine, we were not able to measure any additional persisting inward conductance as a deviation from linearity [20]. This contrasted recently published findings by Sossalla and coworkers [14] who reported ranolazine-sensitive I_Na,late_ for human atrial cardiomyocytes with larger amplitude in AF than in SR, although it must be pointed out that statistical significance of the difference was reached only at 2 Hz and when estimating I_Na,late_ as area under the curve by integrating the time-dependent current.

We have therefore checked the sensitivity of our set-up for detecting I_Na,late_ with ventricular and atrial cardiomyocytes from ΔKPQ mice which clearly exhibit late Na^+^ current [8,22]. Our protocol for human atrial cells (protocol I [20]) was directly compared with a slightly modified protocol used by Sossalla et al. [14] (protocol II, see S1–S3 Figs). In addition, TTX (10 μM) was used to identify current flow through Na^+^ channels. In ΔKPQ cardiomyocytes, TTX-sensitive currents were verified in ventricular and atrial cells, however, current amplitudes were always larger with protocol II than with protocol I. This result is expected, because at 40 mV more negative holding potentials, i.e. -120 mV vs. -80 mV, more Na^+^ channels are available. Neither protocol yielded evidence for I_Na,late_ in cardiomyocytes from WT mice. These results indicate, that I_Na,late_ is clearly detectable in our hands.

### I_Na,late_ in human atrial cardiomyocytes

With TTX as a Na^+^ channel identifying tool I_Na,late_ could also be measured at room temperature with both protocols in human atrial cardiomyocytes, despite of a much smaller amplitude than in murine ΔKPQ myocytes. TTX significantly reduced current at -30 mV in SR and AF cells, and TTX-sensitive current was of significantly larger amplitude in AF than in SR cells. Because of the small size of I_Na,late_ at 250 ms and the slow time course of its decay, current was also analysed at 50 ms. Although this time interval is probably more relevant for the human atrial AP, the technical limitations concerning escape from voltage control during activating I_Na,peak_ are more prominent. During loss of voltage control, Na^+^ channel activation and subsequent inactivation is slower than at defined clamp potentials, and therefore, 50 ms may not be enough time for regaining voltage control, whereas 250 ms is likely to be safe. Although quantification of I_Na,late_ becomes uncertain after escape it is noteworthy that there were significant differences in TTX-sensitive late current between SR and AF.

Protocol II which had been designed by Sossalla et al. [14] to optimize voltage control by activating reduced current amplitude close to the reversal potential of I_Na,peak_ at +50 mV before stepping to the test pulse of -30 mV, could not be applied at 37°C because the cells did not tolerate a holding potential of -120 mV.

At physiological temperature TTX did *not* unmask any I_Na,late_ neither in SR nor in AF cells after 250 ms. Here, TTX-sensitive current was significant when analysed after 50 ms and only in AF. However, because of the even faster kinetics of I_Na,peak_ at 37°C, this finding could be an artifact due to escape of voltage control.

Notwithstanding statistical significance, the absolute amplitudes of TTX-sensitive currents at -30 mV were extremely small, ranging around or even below 10 pA. Average inward current at -80 mV was about ten-fold larger (~90 pA in SR and ~130 pA in AF cells). Since TTX should not affect current amplitude at -80 mV where Na^+^ channels are not active, cells in which current shifted by >10 pA at -80 mV (or >20 pA at -120 mV when there was no pre-pulse to -80 mV) after addition of TTX were not accepted (see S4 – S6 Figs for comparison of results in “selected” and “all data”). The major issue of this comparison is that, although TTX significantly reduces current at -30 mV in all cells (S4 Fig), the statistical significance of the difference between SR and AF cells is lost when cells with unstable holding current are included (see S5 Fig). In ΔKPQ mice, comparison of “selected” versus “all” cardiomyocytes (S6 Fig) shows that selection leads to an underestimation of TTX-sensitive current. The reason for this discrepancy could be that ventricular cardiomyocytes from healthy mice have higher inward rectifier current and are more stable in the first place, so that the selection criteria become arbitrary and will worsen the outcome due to reduction in number.

Taken together, we have confirmed significant TTX-sensitive I_Na,late_ in human right atrial cardiomyocytes at room temperature. This current was larger in cells from AF patients, suggesting that remodeling during persistent AF enhances I_Na,late_. Our study was not intended to characterize the electrophysiological nature of I_Na,late_.

### Effect of ranolazine on human atrial tissue

Ranolazine (30 μM) significantly reduced current amplitude in AF, but was ineffective in SR cells, irrespective of time of analysis. Action potentials from AF trabeculae were more sensitive to ranolazine than APs from SR preparations, i.e. plateau potential PLT_20_ and maximum upstroke velocity dV/dt_max_ were already reduced at 10-fold lower concentrations in AF than in SR. In addition, AF, but not SR APs were significantly prolonged at all ranolazine concentrations.

The ranolazine-sensitive current densities in AF myocytes after 50 ms and 250 ms of Na^+^ channel activation were ~0.14 pA/pF and ~0.09 pA/pF, respectively. The impact of this small current on AF action potentials measured at physiological temperatures is difficult to estimate. Although lowering of PLT_20_ is in accordance with hastening of the repolarization process when persisting inward (depolarizing) current is blocked, this effect can equally well be explained by inhibition of I_Na,peak_ as indicated by reduction of dV/dt_max_ which is a non-linear surrogate parameter for Na^+^ channel availability, i.e. peak Na^+^ current [32,33]. In analogy to the APD-shortening effect due to plateau potential elevation with I_Kur_ blockers [26], the prolongation of APD_90_ by ranolazine could be an indirect consequence of lowering of PLT_20_. In addition, block of hERG K^+^ channels by ranolazine [34] can contribute to APD lengthening. Detailed selectivity analysis of ranolazine block of I_Na,late_ over various other ion currents is available for dog ventricular and atrial cardiomyocytes [17,35], where the IC_50_ value for block of I_Na,late_ (i.e. 5.9 μM) is in the range of therapeutic plasma concentrations (2–6 μM; [17]). However, dog data cannot be directly interpolated to human tissue. We have recently reported that plateau potential and APD_90_ were similarly altered in atrial cardiomyocytes from normal SR and atrial-tachypaced dog hearts, and that dV/dt_max_ was in fact more sensitive to ranolazine in SR than atrial tachypaced canine preparations [36].

Several recent reports suggest that patients with AF may benefit from treatment with ranolazine: Randomized controlled trials showed that treatment with ranolazine reduced the incidence of new-onset AF [37], converted new-onset AF when used in a pill-in-the-pocket approach [38] and was more effective than amiodarone in preventing post-operative AF [39]. These beneficial effects of ranoplazine have been attributed to atrial-selective block of I_Na,peak_ and to block of I_Kr_ [19,35]. Like many other Na^+^ channel blockers [40], ranolazine is considered to block preferentially I_Na,late_ over I_Na,peak_, at least in ventricular cells [17]. Block of I_Na,peak_ is an accepted antiarrhythmic mechanism for treatment of AF but is burdened with ventricular proarrhythmic effects [41], therefore preferential block of I_Na,late_ has been suggested to be an advantageous antiarrhythmic property for suppression of early and delayed afterdepolarizations [19]. It should be noted that under the clinical conditions of the above-mentioned randomized trials, atrial tissue is unlikely to have undergone substantial electrical remodelling, yet in our experiments I_Na,late_ was inhibited by ranolazine only in remodelled cardiomyocytes from patients with persistent AF, suggesting that the drug’s therapeutic effect in non-remodelled atria may not be related to preferential block of I_Na,late_. Other mechanisms of action such as atrial-selective block of I_Na,peak_ due to a more negative potential of half-maximum inactivation in atrial versus ventricular cells [35], frequency-dependent block of I_Na,peak_ and block of I_Kr_, or antioxidant properties are expected to contribute [16]. Although ranolazine inhibits I_Na,late_ with higher potency than I_Na,peak_, it is by no means selective for I_Na,late_ since several other ion channels contributing to the shape of the atrial action potential are blocked as well [19]. Recently, Sicouri et al. reported about highly selective I_Na,late_ blocker GS-458967 [42], however, this drug has not been available to us.

### Expression of sodium channel subunits in SR and AF

Theoretically, a non-inactivating component of Na^+^ current could be related to a fraction of a Na^+^ channel isoform with slow inactivation kinetics due to cellular modulation by for instance phosphorylation [43], or, alternatively, due to different isoforms of the channel with distinct kinetic properties [44]. We have detected, in addition to Nav1.5, β1 and β2, also substantial expression of β4 and to a lower degree β3. Nav1.1.-Nav1.4 as well as Nav1.6 and Nav1.8 were expressed at 100- to 1000-fold lower levels than Nav1.5, confirming that Nav1.5 proteins are the major α-subunits of Na^+^ channels in human atria. Although the mRNA expression level of the transcripts encoding Nav1.5 and all the β-subunits tended to be lower in AF than in SR, the level of significance was not reached. Nav1.2, Nav 1.6, and Nav1.8, however, were expressed at significantly lower levels in AF than SR tissue. The latter two channel isoforms have been associated with late channel openings [45] contributing to I_Na,late_ in mouse and rabbit ventricular cardiomyocytes [46] and were significantly lower expressed in AF than in SR. These expression data render it unlikely that Nav1.6 and Nav1.8 subunits contribute to I_Na,late_ which was larger in AF than in SR.

### Magnitude of I_Na,late_

The contribution of I_Na,late_ to cellular Na^+^ load is controversially discussed [15,47]. Most authors agree that under normal conditions, more Na^+^ enters the cell via the Na,Ca exchanger than via I_Na,late_, but this relationship can change under pathophysiological conditions or at rapid pacing rates [16,47,48]. Sossalla et al [14] reported a current-time integral of ~100 ms·A/F, estimated over 200 ms. Assuming a mean cell capacitance ~100 pF, average current amplitude during 200 ms would be 50 pA, but considerably smaller at the end of the 200 ms pulse because inactivation continues. Assuming that I_Na,peak_ amounts to 10 nA, and assuming that like in ventricular cardiomyocytes I_Na,late_ is less than 0.5–1% of I_Na,peak_ [44], the amplitude of I_Na,late_ should be between 50 and 100 pA, indicating that I_Na,late_ is expected to be of a similar order of magnitude as leak currents.

Though very small in amplitude when compared to I_Na,peak_ [44], I_Na,late_ cannot be neglected, because persistent flow of even a small Na^+^ current during the plateau phase not only prolongs action potential duration but also induces a Na^+^ load that may produce cellular Ca^2+^ overload. Both long action potentials and Ca^2+^ overload are currently accepted mechanisms for arrhythmogenesis in atrial fibrillation [15,49].

## Conclusions

Our findings confirm and extend previously published data about the presence of I_Na,late_ in human atria. Using TTX and ranolazine as tools to expose I_Na,late_, we found in AF myocytes, an average current density of approximately 0.15 pA/pF. This current may contribute to prolongation of early repolarization caused by AF-induced electrical remodelling (triangular AP shape in AF *vs*. “spike-and-dome” morphology in SR). Our results must, however, be interpreted very cautiously since the impact of such small current on human cardiac electrophysiology is unclear. Furthermore, at physiological temperature the difference in I_Na,late_ amplitudes between SR and AF cells did not reach the level of statistical significance. In conclusion, while this study provides evidence for the presence of I_Na,late_ in human atria, the potential of such current as a target for the treatment of AF remains to be demonstrated.

## Supporting Information

S1 Fig Effects of tetrodotoxin on I_Na,late_ measured at room temperature with protocol II in ventricular and atrial cardiomyocytes from ΔKPQ-SCN5A mice. Same lay-out as in Fig 1.
(TIF)

S2 Fig Original recordings of currents in human atrial myocytes. **A** and **B:** Examples of currents recorded with the complete protocol I (**A**) or protocol II (**B**) in human atrial myocytes from patients in sinus rhythm (SR) or atrial fibrillation (AF).
(TIF)

S3 Fig Effects of tetrodotoxin on I_Na,late_ measured with porotocol II in human atrial myocytes at room temperature. **A and B:** Same lay-out as in Fig 2.
(TIF)

S4 Fig Comparison of TTX effects on I_Na,late_ measured with protocol I and II in selected cells (upper row) with results from all cells (lower row), analysis after 50 ms and 250 ms, room temperature. *P < 0.05; **P < 0.01; ***P < 0.001; paired Student’s t-test (comparison between control and drug effect).
(TIF)

S5 Fig Comparison of TTX-(10 μM)-sensitive current and ranolazine-(30 μM)-sensitive current in selected cells (upper row) with results from all cells (lower row), analysis after 50 ms and 250 ms. I_Na,late_ in SR (black) and AF (red) was measured with protocol I and protocol II for TTX, but only with protocol I for ranolazine. Currents are expressed in pA/pF. *P < 0.05; **P < 0.01; ***P < 0.001; paired Student’s t-test (comparison between control and drug effect) or unpaired Student‘s t test with Welch’s correction I (comparison between SR and AF).
(TIF)

S6 Fig Comparison of tetrodotoxin- and ranolazine-sensitive currents in selected cells (upper row) with results from all cells (lower row), analysis after 250 ms. Tetrodotoxin-(10 μM)- and ranolazine-(30 μM)-sensitive currents in human atrial SR and AF cardiomyocytes and tetrodotoxin-(10 μM)-sensitive currents in WT and ΔKPQ mouse ventricular and atrial myocytes measured with both protocols (protocol Ⅰ and Ⅱ) and analysed at -30 mV. *P < 0.05; **P < 0.01; paired Student’s t-test (comparison between control and drug effect) or unpaired Student‘s t test with Welch’s correction I (comparison between SR and AF). Numbers below the columns (x/y) indicate number of cells per number of patients or animals.
(TIF)
