# Supplementary figures and images for: Late Sodium Current in Human Atrial Cardiomyocytes from Patients in Sinus Rhythm and Atrial Fibrillation
Source: PLoS One. 2015 Jun 29;10(6):e0131432. doi: 10.1371/journal.pone.0131432 (PMC4485891; doi:10.1371/journal.pone.0131432)

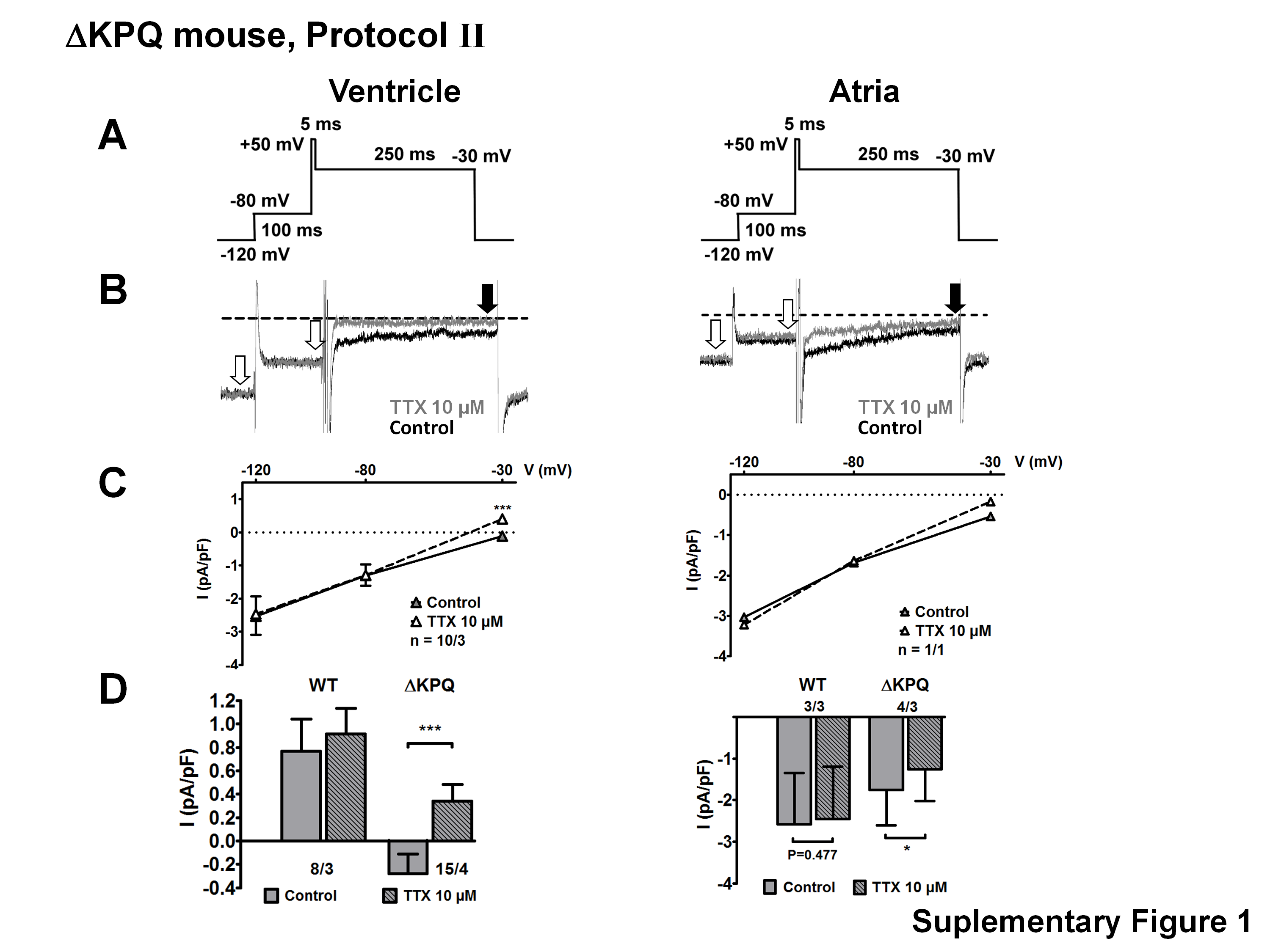

Supplement: S1 Fig — Same lay-out as in Fig 1. (TIF) [file pone.0131432.s001.TIF]

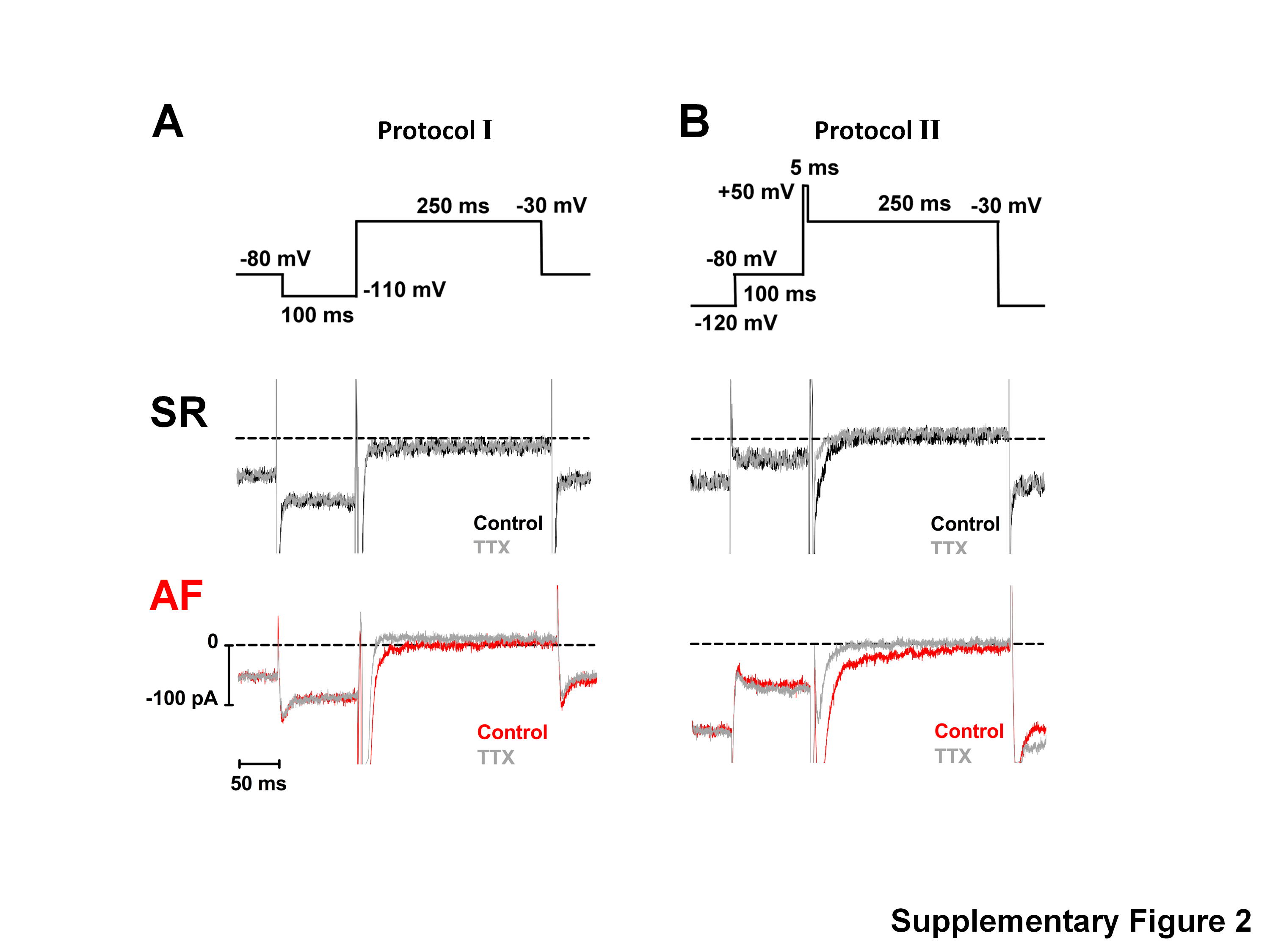

Supplement: S2 Fig — A and B: Examples of currents recorded with the complete protocol I (A) or protocol II (B) in human atrial myocytes from patients in sinus rhythm (SR) or atrial fibrillation (AF). (TIF) [file pone.0131432.s002.TIF]

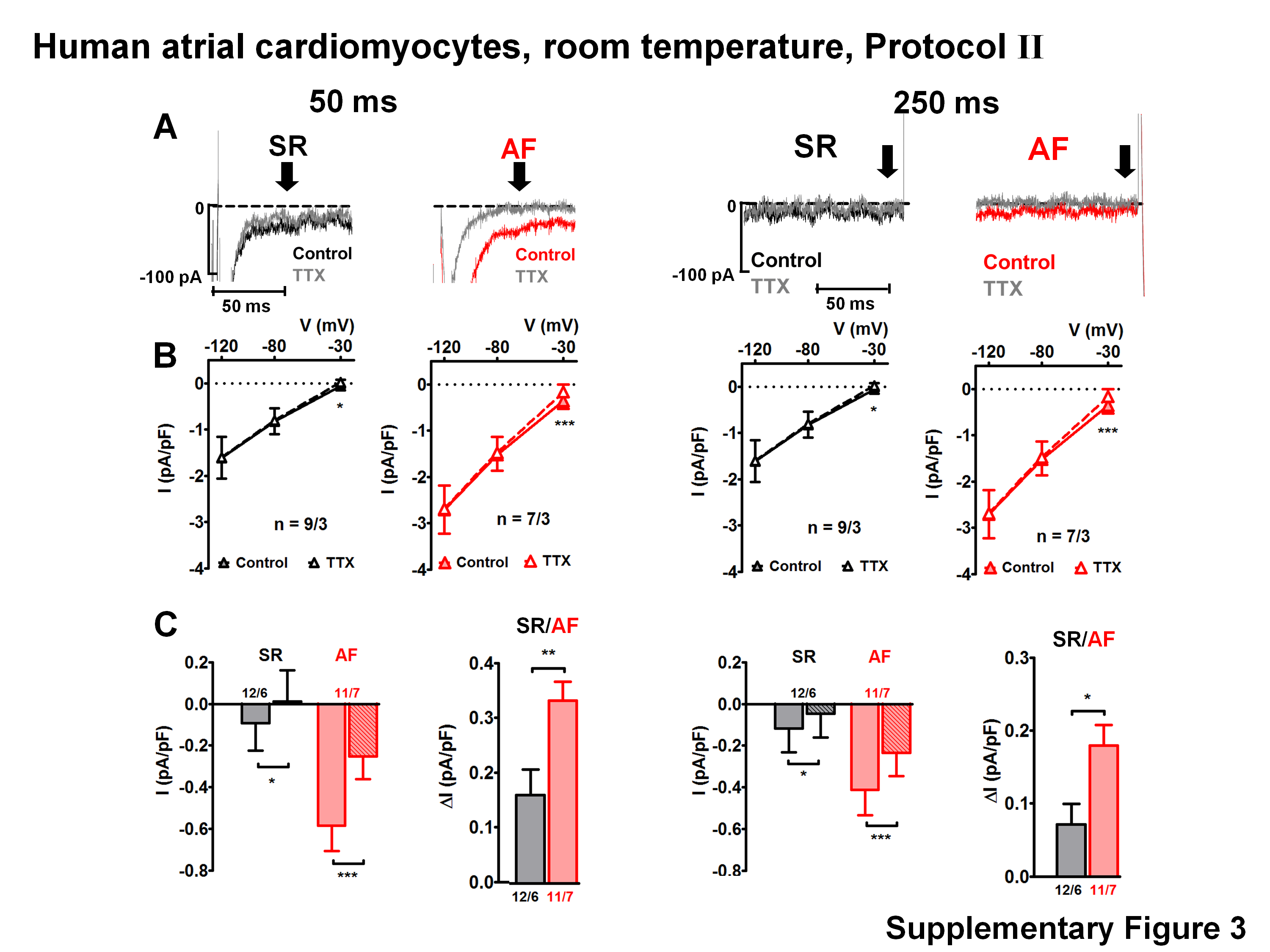

Supplement: S3 Fig — A and B: Same lay-out as in Fig 2. (TIF) [file pone.0131432.s003.TIF]

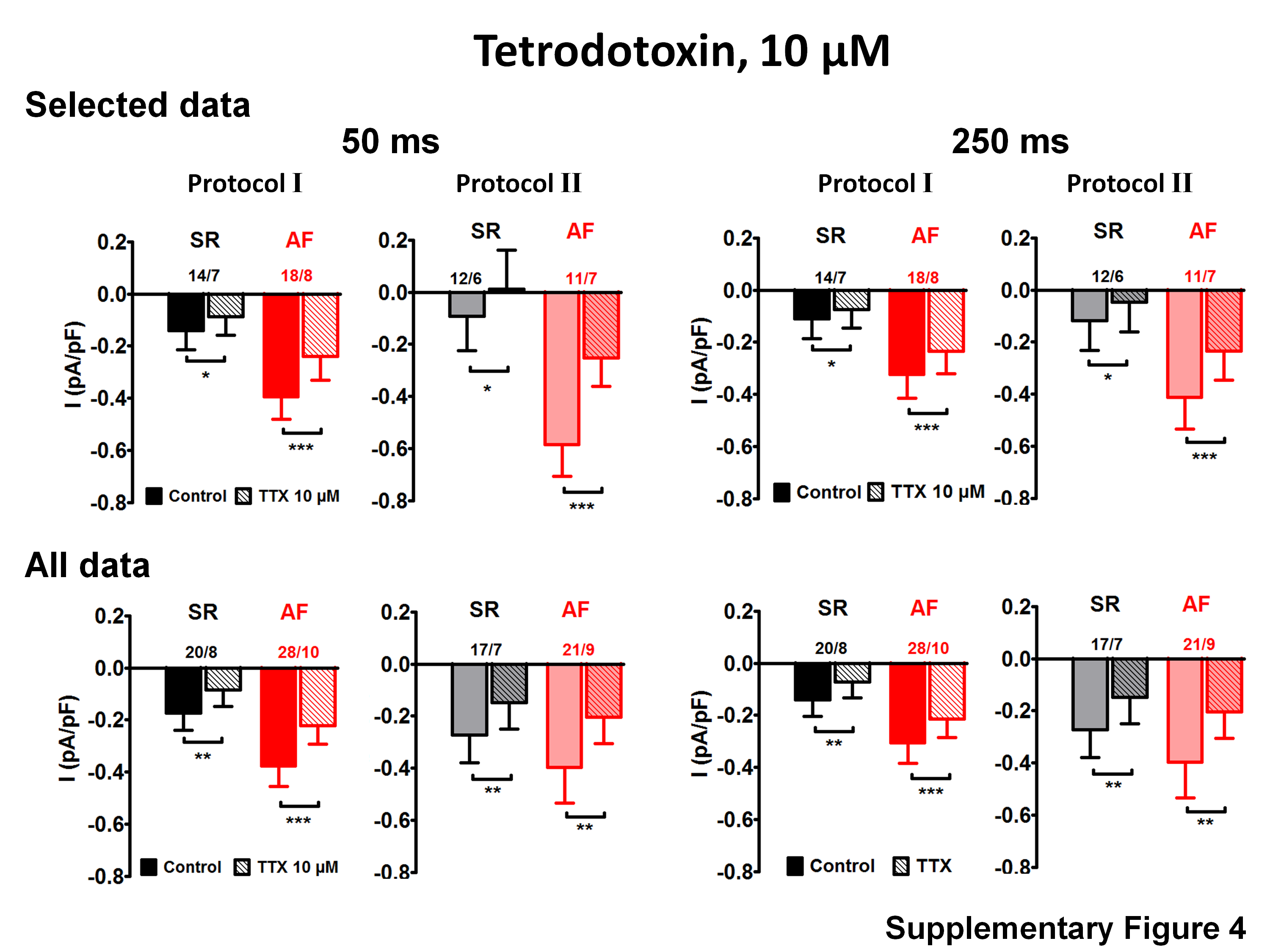

Supplement: S4 Fig — *P < 0.05; **P < 0.01; ***P < 0.001; paired Student’s t-test (comparison between control and drug effect). (TIF) [file pone.0131432.s004.TIF]

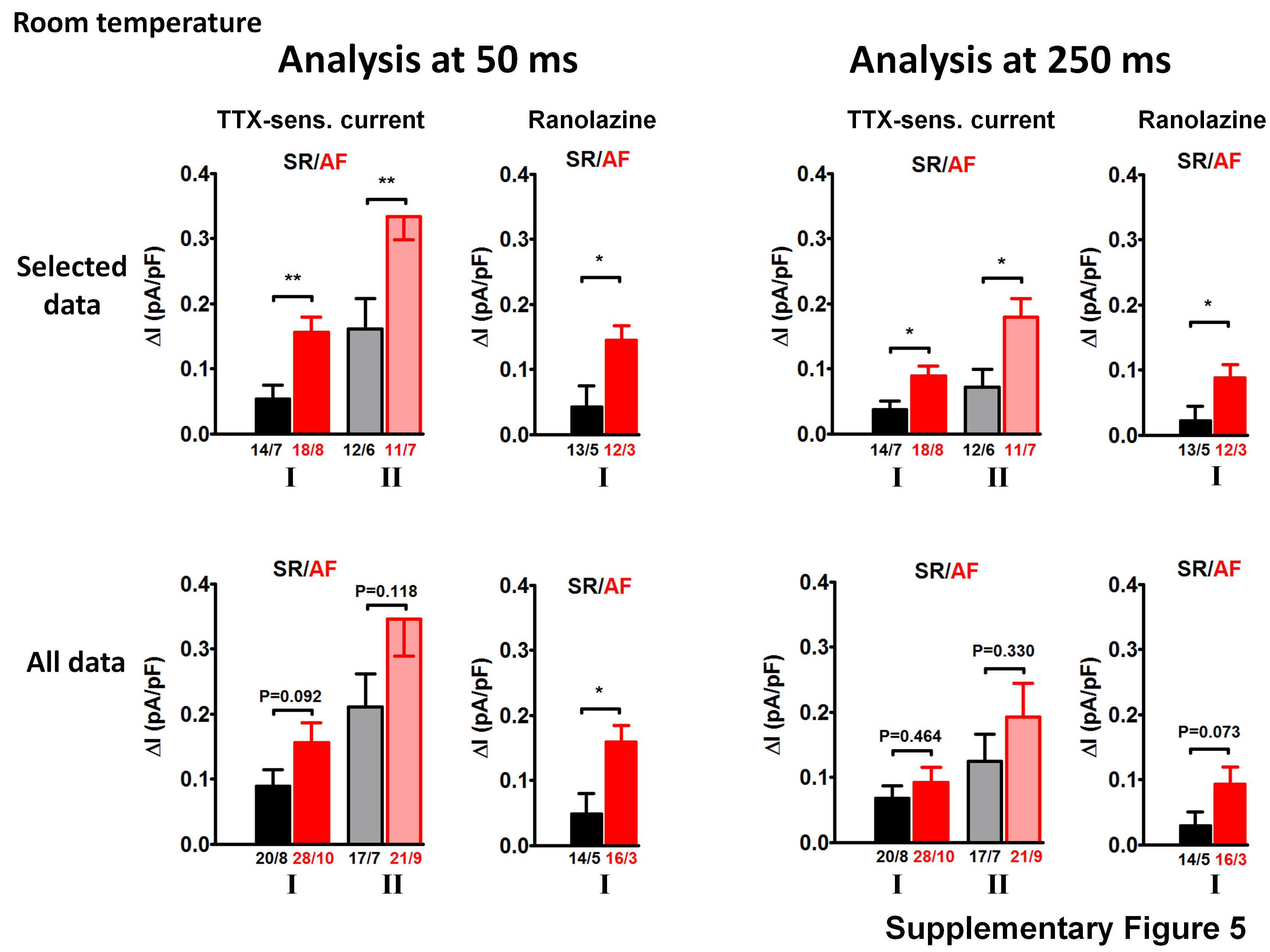

Supplement: S5 Fig — INa,late in SR (black) and AF (red) was measured with protocol I and protocol II for TTX, but only with protocol I for ranolazine. Currents are expressed in pA/pF. *P < 0.05; **P < 0.01; ***P < 0.001; paired Student’s t-test (comparison between control and drug effect) or unpaired Student‘s t test with Welch’s correction I (comparison between SR and AF). (TIF) [file pone.0131432.s005.TIF]

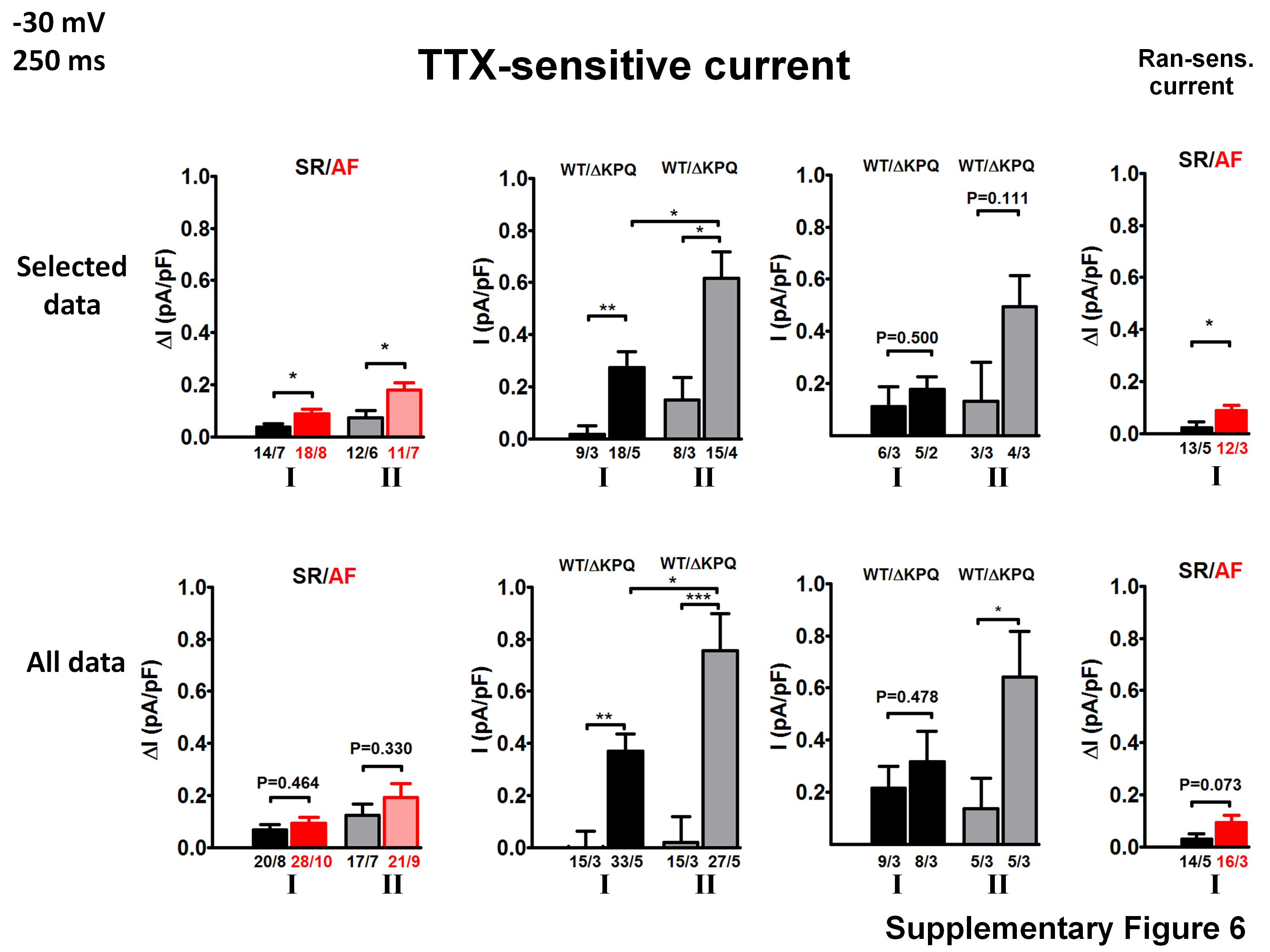

Supplement: S6 Fig — Tetrodotoxin-(10 μM)- and ranolazine-(30 μM)-sensitive currents in human atrial SR and AF cardiomyocytes and tetrodotoxin-(10 μM)-sensitive currents in WT and ΔKPQ mouse ventricular and atrial myocytes measured with both protocols (protocol Ⅰ and Ⅱ) and analysed at -30 mV. *P < 0.05; **P < 0.01; paired Student’s t-test (comparison between control and drug effect) or unpaired Student‘s t test with Welch’s correction I (comparison between SR and AF). Numbers below the columns (x/y) indicate number of cells per number of patients or animals. (TIF) [file pone.0131432.s006.TIF]
